# Is Hemoglobin E Gene Widely Spread in the State of Madhya Pradesh in Central India? Evidence from Five Typical Families

**DOI:** 10.4084/MJHID.2014.060

**Published:** 2014-09-01

**Authors:** R.S. Balgir

**Affiliations:** Department of Biochemistry, Regional Medical Research Centre for Tribals (ICMR), Madhya Pradesh, India

## Abstract

**Background:**

Red cell inherited hemoglobin (Hb) anomalies are commonly encountered in the central region of India. These cause a public health concern due to high level of morbidity, mortality, and fetal loss in the backward, underprivileged, and vulnerable people.

**Purpose:**

To report five typical families of Hb E disorders for the first time detected and identified from various districts of the state of Madhya Pradesh in central India.

**Methods:**

Out of a total of 447 couples/families referred from a tertiary hospital in central India for investigations of anemia/hemoglobinopathies during the period from March 2010 to February 2014, we came across five typical rare couples/families of Hb E disorders (1.1%) worthy of detailed investigations that we have reported here. Laboratory investigations were carried out following the standard procedures after cross checking for quality control from time to time.

**Results:**

For the first time, out of total 27 cases studied, we have encountered nine cases of heterozygous Hb E trait (33.3%), two members (7.4%) with Hb E-β-thalassemia (double heterozygosity), two cases (7.4%) of sickle cell-Hb E disease (double heterozygosity), two β-thalassemia traits (7.4%), three sickle cell traits (11.1%), 9 normal (33.3%), and none with homozygous Hb E disease. Cases of Hb E trait, Hb E-β-thalassemia, and sickle cell-E disease showed moderate to severe anemia, and target cells, and reduced values of red cell indices like red blood cell count, Hb level, hematocrit, mean cell volume, mean cell Hb and mean cell Hb cencentration, describing abnormal hematological profile and clinical manifestations before blood transfusion.

**Conclusions:**

Double heterozygosity of β-thalassemia with Hb S and Hb E is a rare entity, but occurs with severe clinical manifestations, testifying either migrations and/or genetic admixture. Co-occurrence of Hb E/β-thalassemia in different districts indicates that these anomalies along with other hemoglobinopathies are wide spread in Madhya Pradesh and posing a major genetic burden on vulnerable people of central India.

## Introduction

Hemoglobinopathies are characterized by the structurally defective production of hemoglobin (Hb) due to abnormalities in the synthesis of the globin moiety.^1^ Thalassemias are caused by inadequate globin chain production. Hemoglobin - the red pigment in blood cells that changes its structure during human development transfers oxygen to the tissues. Red cell inherited hemoglobin disorders are the commonest monogenic disorders occurring worldwide especially in tropical and subtropical countries including in India.^2^ They are genetically transmitted hematological disorders affecting millions of people. Hb E is the second most globally prevalent hemoglobin variant. 2 It is a slow moving β-chain variant (α_2_β_2_^26^glu>lys) and is common in south-east Asia with allele frequency ranging from 8% to 50–70%.^3^ The cumulative allele frequency of the three most predominant abnormal hemoglobins, i.e. Hb S, Hb E and Hb D has been estimated to be 5.35% in India.^4^,^5^ The prevalence of hemoglobin disorders varies with ethnic group and geographical location in India. Hb E gene is mostly confined to the north-eastern states of Arunachal Pradesh, Assam, Manipur, Meghalaya, Mizoram, Nagaland, Sikkim, Tripura, West Bengal, Jharkhand, and Odisha with the average frequency of 10.9%.^6^–^8^ Sporadic cases have also been reported from other states like Andhra Pradesh, Tamil Nadu, Karnataka, Maharashtra, Gujarat, Uttar Pradesh, etc.

Hb E disorder may encounter in a heterozygous state as E trait, homozygous E disease and compound heterozygous E with other abnormal hemoglobinopathies or β-thalassemia with widely variable clinical phenotype. Most of the known structural variants are harmless but in some cases they may alter the stability or functional properties of the hemoglobin and lead to clinical manifestation. Clinical spectrum of hemoglobin disorders varies from asymptomatic conditions to severe disorders like thalassemia major that requires regular blood transfusions and extensive medical care.^1^,^2^,^9^–^12^ Prospective prevention through carrier detection, antenatal and prenatal screening, and genetic/marriage counseling is the best-possible strategy for prevention and control of these hemolytic disorders.

The clinical features of sickle cell disorders reflect the red blood cells’ propensity to assume a sickle shape in deoxygenated blood, leading to shortened red cell survival and a tendency to block small blood vessels. Even though patients may adapt to their anemia (low level of hemoglobin), their illness is interspersed with acute episodes including the attacks of bone pain; sequestration of blood into the lungs, liver, or spleen; or thrombosis of cerebral vessels, which may cause a stroke.^13^ The patients are extremely prone to infection, particularly during early childhood, and to a wide range of chronic complications.^13^

Since the sickle cell hemoglobinopathy and thalassemias are widely prevalent in tribal as well as nontribal communities in Madhya Pradesh,^14^ we focused the present study on five typical couples/families of hemoglobin E disorders encountered for the first time during the course of screening and investigation for anemia and hemoglobinopathies referred from a tertiary hospital in central India.

## Material and Methods

Suspected couples and their offspring, with at least one suspected/confirmed case of anemia/hemoglobinopathies (homozygous β-thalassemia/HbE/Sickle cell disease or compound heterozygosity), routinely referred by the experts (in Gynecology, Pediatrics, and Blood Bank) to our Centre for confirmation of diagnosis/investigations, and attending the Netaji Subhash Chandra Bose Medical College and Hospital, Jabalpur in Madhya Pradesh, were included in the study. Some personal particulars such as age, caste, marital distance (in Km.), native place, and reproductive history of these couples were also recorded. Out of a total of 447 couples/families referred and investigated for anemia/hemoglobinopathies during the period from March 2010 to February 2014; a total of 200 were found normal, and 247 couples had different hemoglobin disorders (Table 1). Among them we have encountered five (1.1%) couples/families [AE/AA (2), AE/AS (1), AE/β-thalassemia trait (1), SE/β-thalassemia trait (1)] having typical hematological features, variable electrophoretic pattern, and genetic and clinical history worthy of detailed investigations.

Intravenous 2–3 ml blood was taken from each proband under aseptic conditions after taking informed/written consent for screening of hemoglobinopathies and β-thalassemia syndrome. Laboratory investigations were carried out following the standard procedures after cross checking for quality control from time to time. Hematological parameters were studied by using an automated Blood Cell Counter (Model-MS_5_9, Melet Schloesing Laboratories, Cergy-Pontoise Cedex, France).

The sickling test was performed by using 2% freshly prepared sodium metabisulphite solution as reducing agent for the presence or absence of sickle cell hemoglobin.^15^ The routine hemoglobin lysate electrophoresis was carried out on cellulose acetate membrane (CAM) in Tris-EDTA-Borate buffer at pH 8.9 and quantification of A_2_ fraction of adult hemoglobin was done by elution method.^15^, ^16^ The value more than 3.5% of _A2_fraction of adult hemoglobin was taken as a cut off point for determining the β-thalassemia trait. Those individuals having very high hemoglobin A_2_ value, i.e. more than 10% were suspected to have Hb A_2_ plus Hb E; and the analysis was confirmed by the investigations of other family members. Estimation of fetal hemoglobin was done according to the technique described by Weatherall.^16^

The diagnosis of hemoglobin E-β-thalassemia was based on the findings of hemoglobin A, F, E and elevated A_2_ (>3.5%) on electrophoresis under alkaline pH, and cross checked by the laboratory investigations of other family members to differentiate between Hb E and raise Hb A_2_. All the doubtful cases were further subjected to hemoglobin variant analysis for detection of any discrepancy (Bio-Rad Diagnostics, Hercules California, USA).

Results so obtained were given to parents for treatment and further management by the concerned referring doctor. All the carriers/affected persons were given genetic/marriage counseling.^17^

## Results

**Figure 1:**
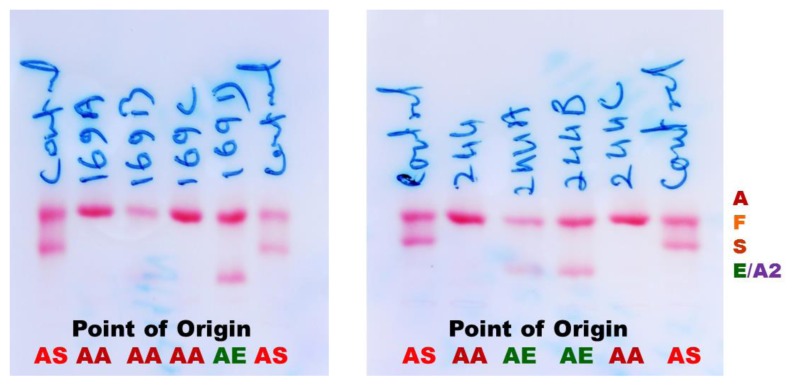
General alkaline electrophoresis pattern on cellulose acetate membrane (AA= Normal Adult Hemoglobin; AS= Sickle Cell Trait; AE= Hemoglobin E)

Table 2 shows two couples/families identified with hemoglobin E gene that was detected in three generations in Madhya Pradesh. These couples/families hailed from Damoh and Jabalpur districts, and belonged to Kalar (serve locally made indigenous alcohol) and Yadav (agricultural cultivator) community, respectively of other backward castes (OBC) of the state. A summary detail of hematological indices and general electrophoretic pattern of hemoglobin AE (Figure 1) is presented in Table 2. Since the electrophoresis was routinely done in our laboratory, hence the pattern was not preserved for these families. For the first time, we have identified six cases of heterozygous hemoglobin E trait (6/9; 66.7%) in these two couples/families.

A summary of typical hematological and genetic features of three other couples/families are presented in Table 3. It may be noted from table 3 that, for the first time from Madhya Pradesh, we have come across two members (2/18;11.1%) with sickle cell-E-disease (double heterozygosity), two members (11.1%) with hemoglobin E-β-thalassemia disease (double heterozygosity), two members (11.1%) with β-thalassemia trait (carrier), three members (16.7%) with hemoglobin E trait (carrier), three members (16.7%) with sickle cell trait (carrier), and six healthy members (33.3%). These families were natives of Dindori, Rewa and Singrauli districts of Madhya Pradesh and belonged to Dhimar (broom makers), Kalar (serve locally made indigenous alcohol), and Teli (who do business in locally prepared mustard oil) castes, respectively (Table 3).

One of the parents from Singrauli district of Madhya Pradesh was suffering from sickle cell-E-disease (Table 3). This scenario reflects ignorance and unawareness about the prevalent hereditary hemolytic disorders in the vulnerable communities of Madhya Pradesh. Average family size was six members, with average children born being 4, indicating no family planning.

It is apparent from Tables 2 and 3 that the majority of the hemoglobin E trait, hemoglobin E-β-thalassemia, and sickle cell-E-disease cases showed moderate to severe anemia, reduced values of red cell indices like red blood cell (RBC), Hb level, hematocrit (HCT), mean cell volume (MCV), mean cell hemoglobin (MCH) and mean cell hemoglobin concentration (MCHC), with the presence of target cells.

In normal as well as in double heterozygous cases, the hematological indices were also found more reduced than the normal standard because of concomitant iron and folic acid deficiency, parasitic infestations and malarial infections. The red cell morphology also showed hypochromia and microcytosis. Both the hemoglobin E-β-thalassemia cases had splenomegaly. The enlarged spleen varied from 2–9 cm below the left costal margin, whereas, the hepatomegaly ranged from 2–4 cm below the right costal margin in these patients. Both double heterozygosity patients reported the history of multiple transfusions ranging from 1–15 units from the age of about one year. The clinical picture in both cases was similar to that of hemoglobin E disease or homozygous β-thalassemia. It is to note that β-thalassemia or Hb E-carrier mothers in their reproductive life either had spontaneous abortions or neonatal deaths. The value of Hb A2 in normal as well as in Hb AS cases remains in between 1.5 to 3.5% range, and beyond 3.5% it is labeled as β-thalassemia trait. Simultaneously, the region of central India falls within the hyperendemic site for malaria and persons inhabiting in the region are immunological sensitized against it, that may in some cases raise the Hb A2 level. However, under exceptional situation this normal range exceeds 3.5%; therefore, family studies/molecular analysis of mutations are required as reported by Yang et al.^18^

An interesting point emerges out from the present study is that these double heterozygosis parents have married for convenience within the radius of less than 40 km. away only from their native place (Table 2 and 3).

## Discussion

Although hemoglobin disorders are of a worldwide occurrence, yet some communities and geo-ecological regions have high prevalence of specific hemoglobin variants either due to the practice of consanguinity or natural selection against malaria.^7^ The inherited disorders of hemoglobin synthesis are one of the important public health challenges in several parts of India.^7^,^19^ The north eastern region of India being hyper-endemic for malaria, is a huge reservoir of hemoglobin E variant and β-thalassemia,^7^ whereas, the sickle cell disease constitutes a sickle cell belt in central India.^14^ The north-western region predominates with β-thalassemia, hemoglobin D variant and compound heterozygosity of various other known variants and thalassemias.^20^,^21^ The findings of occurrence of hemoglobin E gene are one of the most fascinating biological interactions of the people in central India.

For the first time, we have identified several members of the families with Hb E and double heterozygosity. Out of 27 cases, we have encountered nine cases of heterozygous hemoglobin E trait (33.3%), two members (7.4%) with hemoglobin E-β-thalassemia (double heterozygosity), two cases (7.4%) of sickle cell-hemoglobin E disease (double heterozygosity), two β-thalassemia traits (7.4%), three sickle cell traits (11.1%), 9 normal (33.3%), and none with homozygous hemoglobin E disease (Tables 2 and 3) in the state of Madhya Pradesh in central India. It is an important aspect of disabling disorders of sickle cell disease and β-thalassemia syndrome that the practices of community genetics, endogamy, and consanguineous marriages have an impact on neonatal and infant health.^21^ In populations where consanguineous marriage is widely practiced, recessive genetic disorders continue to gain greater prominence in the overall spectrum of ill health.^22^ Practice of the community endogamy brings less variety in the population because same normal or abnormal genes in combination multiply devoid of any change in DNA recombination in the community. In addition, endogamy and consanguineous marriages lead to inbreeding, consequently, increasing the homozygosity (homozygosis) of recessively inherited deleterious genetic traits such as homozygous sickle cell disease, homozygous hemoglobin E disease or β-thalassemia major. This lowers the fitness of mating parents or of the population.^22^,^23^ The practice of communities to marry for convenience within the radius of less than 40 km. away from their native place amounts to inbreeding and increases homozygosity of the defective genes. Thus, outcome of carrier parents of these disorders is disastrous one as revealed by the present study.

Evidence from historical perspectives^24^,^25^ revealed that with the establishment of British East India Company in 1757 at Kolkata in Bengal and the subsequently proliferation and capture of significant administrative and political authorities in India led to appointments of local administrative manpower and subordinate staff and led to the establishment of educational institutes for furtherance of company’s interests. During British regime/rule for about 200 years in India, these Bengalee origin recruits who were the reservoirs of abnormal hemoglobin E and β-thalassemia gene, might have misused or abused administrative (powers) authority (being holding the high positions) to exploit the local inhabitants wherever these people were posted outside Bengal for their vested biological interests and might have infiltrated the abnormal hemoglobin E and β-thalassemia genes in the local community and vice versa. Some of these people had also permanently settled in other states of India. As a result of permanent settlements outside Bengal under British rules in India at that time, and infiltrations and migrations of abnormal genes sporadically spread in almost each and every state of India especially in different towns and cities due to intercaste/interstate matrimony, in some cases, with the passage of time; and the abnormal hemoglobin E or β-thalassemia genes are now encountered depending upon the intensity of penetration in the population. In this fashion, in concurrence with other states of India, the state of Madhya Pradesh was not an exception to this process. However, our verbal enquiry for tracing the migration of the studied families (Tables 2 and 3) from/to West Bengal or north eastern region of India for marriage alliance with Bengalee partner did not give any positive clue for the infiltration of the abnormal gene (s) because several generations since have been passed and the young people of the present generation might not be aware of the past reproductive history of their fore-fathers/mothers.

The findings of the present study are consistent with the infiltration of hemoglobin E gene and migrations of the people from north-eastern region of India especially from the West Bengal and Odisha states because such cases with hemoglobin E and β-thalassemia genes are encountered in the respective other states of India such as Odisha,^8^,^26^ Uttar Pradesh,^10^ Tamil Nadu,^11^ Gujarat,^12^ Maharashtra,^21^ Karnataka,^27^ Andhra Pradesh,^28^ etc. which are referred to the local hospitals. For example, a hospital based study^12^ in Gujarat state has shown the high prevalence of hemoglobin E gene because an enormous labor force from Odisha state has migrated to Surat City (a Textile hub) harboring the abnormal hemoglobin E gene. Similar hospital based findings were reported from Tamil Nadu^11^ and Uttar Pradesh.^10^ Our findings get further support and strength from a molecular study carried out from Gwalior-Chambal region of Madhya Pradesh in central India, where Hb E alleles were reported to be 10.83 percent.^29^ It is interesting to note that out of β-thalassemia mutations mostly prevalent in India, the IVS-1, 5 (G→C) mutation has the highest frequency of 72% in Eastern India^30^ with the exception of Southern India (85%) where consanguinity is highly practiced, and its frequency of 56.67% in Gwalior-Chambal region^29^ and 39.5% in and around Bhopal^31^ of Madhya Pradesh has so far been reported in central India. Among the common β-thalassemia mutations so far identified from central India are reported here: IVS1, 1(G →T), 619bp Del, Cap+1(A → C), Codons 8/9(+G), Codons 41/42 (-TCTT), and Codon 30 (G→ C).^29^,^31^

It is apparent from the present study that the patients of hemoglobin E-β-thalassemia and sickle cell-β-thalassemia disease manifest heterogeneity in clinical manifestations, hematological picture, prognosis and management profile in the state of Madhya Pradesh (Tables 2 and 3). The patients with early onset and severe anemia have the disease course similar to homozygous β-thalassemia in the former and that of sickle cell disease in the latter case, while those patients with late onset with mild hemolytic anemia manage with the occasional blood transfusions or remain completely asymptomatic without any hemolytic crisis in life. Similar observations were made for Hb E-β-thalassemia in Thailand^3^ and Sri Lanka^32^ and for sickle cell-β-thalassemia in India.^2^,^8^,^13^,^33^

The severity of phenotype is, therefore, dependent on the type of β-thalassemia mutation, depending upon the levels of Hb E and Hb F and the number of β-globin genes, which tend to reduce the severity of the disease.^2^,^13^ The interaction of hemoglobin E and β-thalassemia results in a broad spectrum of clinical conditions, in some cases indistinguishable from β-thalassemia major, whereas, in others it has a milder course without dependent on transfusion. These findings get further support from the earlier studies.^2^,^8^,^13^ The Hb E is a mild structural variant of β-globin chain, being asymptomatic in homozygous state.^8^ The clinical presentations of all the cases of Hb E-β-thalassemia were identical with those of β-thalassemia major patients in Madhya Pradesh. The peripheral blood smear examination revealed a hypochromic and microcytic picture with predominance of target cells. All the patients in the present study were transfusion dependent from the age of one year onward and needed multiple blood transfusions, varying from 1 to 15 units.

All the patients had pallor, fatigue, recurrent fever, joint pains, splenomegaly and hepatomegaly of varying degree or grades. The clinical picture is compatible with the studies reported from other parts especially from the North-Eastern region of India where its frequency is reported to be very high.^7^ Severely affected patients of Hb E-β-thalassemia had marked anemia, jaundice, bossy maxillary bones and prominent hepatosplenomegaly. Apparently we could not notice any visible as well as palpable hyperthyroidism in sickle cell anemia, SE or and E-β-thalassemia in our series of patients (as reported by Yang et al.^18^). Further, mutational analysis could provide some clues in this direction.

The situation of inherited hemoglobin disorders in general is very grim in India^5^ and particularly grave in Madhya Pradesh. There are a large number of families who are genetically afflicted and who have to bear the brunt of clinical manifestations of sickle cell/Hb E disorders and β-thalassemia syndrome. The vulnerable people are not aware of these hereditary health problems, neither their mode of transmission and available testing facilities, and not to talk of their knowledge about remedies.^17^ Primary Health Centres (PHCs) are not adequately equipped with necessary infrastructure and trained staff to tackle the emerging emergencies of blood transfusion and treatment for the affected people.

Once the infrastructural facilities are established in Medical Colleges and Hospitals or at Primary Health Centre levels by the state government, it would be convenient to diagnose and identify the carriers and the genetic/marriage counselors would be able to explain to the parents about the general supportive measures like use of oral rehydration solution, drinking lot of liquid, juice or water, avoiding adverse climatic conditions, etc. complete immunization, prophylactic dose of penicillin and pneumococal vaccine may avoid repeated infections. Prenatal diagnosis is one of the important aspects of preventive program of genetic disorders or birth defects.^5^,^8^

Thus, it is apparent from the distribution of hemoglobin E and β-thalassemia in the encountered couples/families from different districts of Madhya Pradesh (Table 2 and 3) that this abnormal hemoglobin E gene has wide spread in central India.

## Figures and Tables

**Table 1:** Spectrum of hemoglobinopathies in couples referred to a tertiary hospital in Central India.

Spectrum of Hemoglobinopathies in Couples	No. of Couples (N)	Percent	Interpretation of genotypes
**AA/AS**	**65**	**14.54**	Normal husband and sickle cell trait wife or normal wife and sickle cell trait husband
**AA/SS**	**18**	**4.03**	Normal husband and sickle cell disease wife or vice versa
**AS/AS**	**72**	**16.11**	Both husband and wife are carrier for sickle cell disease
**AS/SS**	**9**	**2.01**	One partner is carrier for sickle cell disease and other partner is suffering from sickle cell disease
**AS/Sickle cell-β-Thalassemia**	**4**	**0.90**	One partner being carrier of sickle cell disease and the other partner is sickle cell-β-thalassemia (having compound disease, i.e. sickle cell disease and β-thalassemia)
**β–Thalassemia Trait/Sickle cell β-Thalassemia**	**4**	**0.90**	Stands for one partner being carrier for thalassemia major and the other counterpart is sickle cell-β-thalassemia
**AA/Sickle cell-β–Thalassemia**	**3**	**0.67**	Stands for one partner being normal and the other partner is sickle cell-β-thalassemia
**AS/β–Thalassemia Trait**	**15**	**3.36**	Stands for sickle cell carrier husband and β-thalassemia trait wife or vice versa
**AA/β–Thalassemia Trait**	**17**	**3.80**	Denotes for normal husband and β-thalassemia trait wife or normal wife and β-thalassemia trait husband
**β–Thalassemia Trait/β–Thalassemia Trait**	**35**	**7.83**	Denotes that both husband and wife are carrier for thalassemia major
**AE/AA**	**2**	**0.45**	Stands for one partner being normal and the other one is hemoglobin E trait
**AE/AS**	**1**	**0.22**	Stands for one partner being a sickle cell trait and the other one is hemoglobin E trait
**AE/β–Thalassemia Trait**	**1**	**0.22**	Stands for one partner carrier of hemoglobin E disease and the other partner is carrier of thalassemia major
**SE/β–Thalassemia Trait**	**1**	**0.22**	Denotes that one partner is carrier of thalassemia major and the other partner is suffering from a compound disease of hemoglobin E and sickle cell trait
**Hemoglobinopathies**	**247**	**55.26**	Combined all types of hemoglobinopathies
**AA/AA (Normal)**	**200**	**44.74**	Normal husband and normal wife (control)

**Table 2:** Two families with hemoglobin E gene in Madhya Pradesh state of Central India

District	Jabalpur					Damoh			
Caste	Yadav (Other Backward Caste)					Kalar (Other Backward Caste)			
Marital Distance in Km.			40				25		
Relationship	Maternal Grand Father	Maternal Grand Mother	Father	Mother*	(Proband) Son	Grand Mother	Father	Mother **	(Proband) Son
Age in years	42	39	27	22	3	47	25	20	2
Sex (Male/Female)	M	F	M	F	M	F	M	F	M
Hb (g/dl)	14.46	11.80	14.22	8.43	10.54	10.20	10.90	10.34	10.60
RBC(x10^12^/l)	5.97	5.10	5.43	4.01	5.41	5.51	6.46	5.34	4.66
MCV(fl)	77.89	69.65	77.18	59.62	46.65	57.91	49.40	78.20	62.49
HCT (%)	46.50	35.52	41.91	23.91	25.24	31.91	31.91	41.76	29.12
MCH(pg)	24.22	23.14	26.19	21.02	19.48	18.51	16.87	19.36	22.75
MCHC (g/dl)	31.10	33.22	33.93	35.26	41.76	31.96	34.16	24.76	36.40
WBC (x10^12^/l)	5.75	6.71	5.81	5.51	11.90	5.71	5.50	5.55	4.86
RDW (%)	15.50	15.32	14.40	15.30	11.74	15.10	15.06	14.81	13.22
Sickling	−ve	−ve	−ve	−ve	−ve	−ve	−ve	−ve	−ve
Electrophoresis (pH 8.9)	AA	AE	AA	AE	AE	AE	AE	AA	AE
Hb A_2_/E (%)	2.90	26.71	2.64	26.63	27.10	27.53	29.91	2.33	28.62
Hb Fetal (%)	0.74	1.34	0.82	1.24	0.93	1.84	1.23	1.12	1.34

AA=Normal Adult Hemoglobin; AE=Hemoglobin E Trait

* Age at menarche 14 years and at present carries six months pregnancy.

** Age at menarche 15 years and at present carries eight months pregnancy.

**Table 3:** Three families with hemoglobin E gene in Madhya Pradesh state of Central India

District	Dindori			Singrauli						Rewa								
Caste	Dhimar (Scheduled Caste)			Teli (Other Backward Caste)						Kalar (Other Backward Caste)								
Marital Distance	8 Km			3 Km						30 Km								
Relationship	Father	Mother	Son	Father	Mother	D-1	D-2	D-3	Son-4	Father	Mother	D-1	D-2	Son-3	D-4	D-5	Son-6	D-7
Age in years	31	28	9	45	39	23	20	15	12	45	40	25	23	19	17	15	11	5
Sex (Male/Female)	M	F	M	M	F	F	F	F	M	M	F	F	F	M	F	F	M	F
Hb (g/dl)	11.50	10.41	6.03	9.92	10.01	10.62	5.72	10.21	9.82	12.01	8.82	12.71	11.61	11.51	2.91	10.71	10.21	10.61
RBC(x10^12^/l)	5.33	5.70	2.92	3.73	5.12	3.63	2.97	5.54	3.91	5.59	4.90	4.72	4.35	4.77	1.54	4.62	4.20	4.22
MCV (fl)	64.73	57.09	67.53	74.29	62.52	87.66	63.39	56.52	74.50	64.42	54.08	77.35	79.77	79.04	62.47	77.27	79.07	77.96
HCT (%)	34.50	32.54	19.72	27.71	32.01	31.82	18.80	31.31	29.13	36.01	26.50	36.51	34.70	37.70	9.62	35.70	33.21	32.90
MCH (pg)	21.57	18.26	20.65	26.59	19.55	29.26	19.26	18.43	25.11	21.48	18.00	26.93	26.69	24.13	18.90	23.18	24.31	25.14
MCHC (g/dl)	33.33	31.99	30.58	35.80	31.27	33.37	30.42	32.61	33.71	33.35	33.28	34.81	33.46	30.53	30.25	30.00	30.74	32.25
RDW (%)	12.42	14.31	12.03	14.61	14.41	13.81	16.71	13.10	12.62	12.02	14.02	13.90	13.40	13.41	15.50	14.70	12.61	10.31
WBC(x10^12^/l)	4.54	5.40	6.22	5.90	4.82	5.70	5.72	5.81	5.81	5.41	5.20	5.71	5.90	5.41	4.81	5.12	5.22	5.90
Sickling	+ve	−ve	+ve	+ve	−ve	+ve	−ve	−ve	+ve	−ve	−ve	−ve	−ve	−ve	−ve	−ve	−ve	−ve
Electrophoresis (pH 8.9)	AS	AE	SE	SE	AA_2_	AS	EF	AE	AS	AE	AA_2_	AA	AA	AA	EF	AA	AA	AA
Hb A_2_/E (%)	2.31	26.71	-	-	5.22	2.62	67.42	27.12	2.31	24.61	4.23	3.02	3.22	2.32	48.41	2.90	2.61	2.71
Hb Fetal (%)	0.35	1.24	2.52	2.62	1.23	1.12	6.55	1.03	0.80	1.62	0.45	0.61	0.80	0.51	13.40	0.72	1.02	1.23

D= Daughter; AA= Normal Adult Hemoglobin; AS= Sickle Cell Trait;

SE= Sickle Cell-E Disease; AA_2_ = β-Thalassemia Trait; AE= Hemoglobin E Trait

EF= E-β-Thalassemia Disease
